# An investigation of association between human milk mineral patterns and infant growth

**DOI:** 10.3389/fnut.2024.1387956

**Published:** 2024-06-19

**Authors:** Han Sun, Qinggang Xie, Yalin Zhou, Yang Liu, Jiancun Pan, Yajun Xu, Shilong Jiang, Kaifeng Li

**Affiliations:** ^1^Feihe Research Institute, Heilongjiang Feihe Dairy Co., Ltd., Beijing, China; ^2^PKUHSC-China Feihe Joint Research Institute of Nutrition and Healthy Lifespan Development, Beijing, China; ^3^Department of Nutrition and Food Hygiene, School of Public Health, Peking University, Beijing, China; ^4^Beijing Key Laboratory of Toxicological Research and Risk Assessment for Food Safety, Peking University, Beijing, China

**Keywords:** human milk, mineral, cluster, metabolomics, infant z score, CHMP

## Abstract

**Introduction:**

Human milk is widely acknowledged as the optimal food for infant aged 0 ~ 6 months. While there has been extensive documentation on the mineral and trace element composition of human milk, results on the relationship between mineral content and infant growth remain mixed. This cross-sectional study aims to explore human milk mineral patterns and to investigate associations between human milk mineral patterns, human milk metabolomic profile and infant growth.

**Methods:**

A total of 200 breast milk samples from seven cities in China was included. Human milk mineral and trace elements was detected by inductively coupled plasma mass spectrometer (ICP-MS). K-means cluster analysis was utilized to derived human milk mineral patterns. Untargeted human milk metabolomic profiles was determined using high performance liquid chromatography–tandem mass spectrometry (HPLC–MS/MS). Differences of infant growth rate and metabolomic profiles were then compared across patterns identified.

**Results:**

Three human milk mineral patterns were identified. Cluster I was characterized as the highest levels of potassium, magnesium and calcium, while the lowest levels of copper, zinc, manganese and selenium. Cluster II showed the most abundant sodium, iron, zinc, manganese and selenium. Cluster III had the lowest levels of sodium, potassium, magnesium, iron and calcium. Infants of cluster I showed significantly higher length-for-age z score (0.60 ± 2.03, *p* = 0.03). Compared with other clusters, samples of cluster I showed lower expression of metabolites of arachidonic acid (ARA) and nicotinate and nicotinamide metabolism pathway.

**Discussion:**

A human milk mineral pattern was identified which is related to increased infant growth rate and altered metabolic signature. Future work is needed to understand these human milk patterns in terms of biologic mechanisms and generalization to other populations.

## 1 Introduction

Human milk is generally considered to be the optimal food for infant aged 0 ~ 6 months. Minerals are indispensable nutrients for the human body, playing a pivotal role in the growth and development of infants and young children (1). Previously, human milk mineral and trace elements composition have been extensively documented in Chinese (2–5) and other populations (6–8). For example, mean levels of zinc decreased from 3.9 mg/kg in transitional milk to 1.3 mg/kg in mature milk in a Chinese cohort, while concentration of iron remains constant across lactation stages with a mean concentration around 1.0 mg/kg (5). These comprehensive understanding of human milk mineral is crucial for infant formula to further mimic compositional and functional aspect of human milk.

However, a recent systematic review showed that association between human milk mineral content and child growth was generally mixed (9). One possible reason for this may be the presence of complex interplay of human milk mineral composition, which may lead to substantial unexplained variance in the infants (10). Conventional methods reliance on analysis of single variable are therefore not sufficient to capture such metabolic heterogeneities (11). Interestingly, one study applied principal component analysis (PCA) to derive mineral patterns from Guatemalan human milk, and two mineral patterns were significantly associated with infant weight for age z scores (WAZ) and length for age z scores (LAZ) (8). However, maternal habitual dietary intake was not assessed in this study, which makes it impossible to identify any dietary origin of human milk mineral patterns. Moreover, human milk component (such as metabolomics) other than minerals was not determined. Evidence have shown that minerals are linked to various metabolic pathways involving fatty acids and amino acids in the human body. It is therefore important to analyze the differentiations within these metabolic pathways to fully understand the mechanisms underlying the impact of minerals on infant growth.

Against this backdrop, the objective of the present study was to identify patterns of mineral and trace elements compositions in Chinese breast milk using k-means cluster analysis. And to analyze relationships between different clustering characteristics, maternal dietary intakes, infant anthropometric outcomes from a metabolic pathway perspective.

## 2 Methods

### 2.1 Analysis samples

This study is based on the Chinese Human Milk Project (the CHMP study), which is a cross-sectional, multi-regional study involving over 1,800 mother-infant dyads aimed at investigating the characterization and influence factors on Chinese human milk (NCT03675204). The CHMP was approved by the review board of Shanghai Nutrition Society Ethic Committee (Ethic Approval [2016] No. 006). Informed written consent was obtained from all participants. The descriptions of inclusion/exclusion criteria and participants flow chart is displayed in Supplementary Figure S1. Based on this criterion, a total of 200 breast milk samples from seven cities across China’s south, north, inland, coastal, Central Plains, southeast and northwest regions with the lactation stages of 2nd and 6th month were randomly selected for this study. Participants were instructed to collect an HM sample between 9:00 and 11:00, with a minimum of 1.5 h since the previous morning lactation session. One breast was fully expressed using an electric pump. The milk that was collected was then carefully measured using a calibrated scale. The milk was gently mixed to ensure homogenization and then divided into 6 × 5 mL tubes; any extra samples were divided into 10 mL tubes as needed. Aliquoted samples were delivered to our laboratory using dried ice and subsequently stored at −80°C. The detailed sample collection protocols are available elsewhere (12).

### 2.2 Maternal and infant characteristics

Maternal body weight and height were measured upon sampling by calibrated electronic scales while participants were only wearing indoor clothing and no shoes. Weight and length of infant were measured using a tared scale and a length board with a sliding foot piece, respectively. LAZ, WAZ, and weight-for-head circumference z scores (HCZ) were calculated based on the WHO Child Growth Standards (13).

### 2.3 Determination of human milk minerals

The determination of human milk (HM) minerals was carried out by Shanghai Biotree Biotech Co., Ltd according to the National Standard (14). Individual human milk sample was accurately weighed (4–5 g, accurate to 0.0001 g), and placed in a polytetrafluoroethylene crucible that had been pretreated with acid. Samples containing ethanol or carbon dioxide were heated on a hot plate at a low temperature to evaporate these substances. Subsequently, 10 mL of nitric acid-perchloric acid mixture (v/v = 10:1) (Ammonium nitrate and perchloric acid were provided by Sinopec Nanjing Chemical Industries Co. Ltd., GR.) was added for ablation. As soon as the ablation solution turned brownish-black, a small amount of mixed acid was added. The solution was cooled once white smoke appeared and the solution became transparent or slightly yellow. The final solution volume was adjusted to 10 with 1% nitric acid. The test solution was then used to determine the content of each element. A full-procedure blank test was included to check for elemental residues in the pre-treatment vessels and reagents. The blank data indicated almost no residue of Zn. The element contents of the test solution were then determined using an inductively coupled plasma mass spectrometer (ICP-MS, ICAP6300 Thermal, USA). The calibration curve for the measured substance is plotted by using the determinands as the horizontal axis and the ratio of their reaction signal to that of an external standard element as the longitudinal axis. The content of the measured substance in the digestion solution was then obtained by using the standard curve. The main operating parameters of the instrument are shown in Supplementary Table S1 and the detection and quantification limits (LOD and LOQ) are shown in Supplementary Table S2.

### 2.4 Estimation of maternal dietary intakes

A food frequency questionnaire (FFQ) included in the Supplementary Tables S3, S4 was used to estimate maternal dietary intakes and specific types of nutritional supplements consumed. The FFQ includes cereal, coarse grain, starchy roots, dark-colored vegetables, fruits, light-colored vegetables, soybean and soybean products, nuts, egg, meat and meat products, dairy products, fish and aquatic products, nutrition supplements, cooking oil and savory. For each food item, participants were asked to report the frequency of habitual consumption (daily, weekly, monthly, or never) and the amount consumed over the past 1 month. We extracted information of whether individual consumed each food group or not during the past month.

### 2.5 Untargeted metabolomic analysis

#### 2.5.1 Metabolites extraction

Metabolites were extracted by mixing 100 μL of sample and 400 μL of extract solution [acetonitrile: methanol = 1:1, containing mixture of three isotopically labeled internal standards (IS)]. The IS was carefully selected to avoid any potential intervene with HM endogenous metabolism. The mixture was vortexed for 30 s, sonicated for 10 min in ice-water bath, and incubated for 1 h at −40°C to precipitate proteins. The mixture was then centrifuged at 12000 rpm for 15 min at 4°C. The resulting supernatant was transferred to a fresh glass vial for analysis. Quality control (QC) samples (21 QC in total) were prepared by mixing an equal aliquot of the supernatants from all the samples.

#### 2.5.2 LC–MS/MS analysis

LC–MS/MS analyses were performed using an UHPLC system (Vanquish, Thermo Fisher Scientific) with a UPLC BEH Amide column (2.1 mm × 100 mm, 1.7 μm) coupled to Q Exactive HFX mass spectrometer (Orbitrap MS, Thermo). The mobile phase consisted of 25 mmol/L ammonium acetate and 25 ammonia hydroxide in water (pH = 9.75) (A) and acetonitrile (B). The auto-sampler temperature was 4°C, and the injection volume was 3 μL. The QE HFX mass spectrometer was used for its ability to acquire MS/MS spectra on information-dependent acquisition (IDA) mode in the control of the acquisition software (Xcalibur, Thermo). In this mode, the acquisition software continuously evaluates the full scan MS spectrum. The ESI source conditions were set as following: sheath gas flow rate as 30 Arb, Aux gas flow rate as 25 Arb, capillary temperature 350°C, full MS resolution as 60,000, MS/MS resolution as 7,500, collision energy as 10/30/60 in NCE mode, spray Voltage as 3.6 kV (positive) or − 3.2 kV (negative), respectively.

#### 2.5.3 Data preprocessing and annotation

The raw data were converted to the mzXML format using ProteoWizard and processed with an in-house program, which was developed using R and based on XCMS (15), for peak detection, extraction, alignment, and integration. Then an in-house MS2 database built with over 2,000 chemical standards was applied in metabolite annotation. The similarity score of MS/MS spectra was calculated by using the forward dot-product algorithm (15). The cutoff for annotation was set at 0.8, with 248 metabolites being identified from 200 breast milk samples.

#### 2.5.4 Statistical analysis

R (version 4.2.2)^1^ was utilize for all data analysis and producing graphs. The *NbClust* R package, based on K-means clustering algorithm, was employed to determine the optimal number of clusters in the datasets. K-means cluster analysis was used to derive possible human milk mineral patterns. Twenty-four indices were used to decide the optimal number of clusters according to the majority rule, which were integrated in the *NbClust* R package (version 3.0.1.) (16). Multiple linear regressions adjusted for infant age, infant sex and city were carried out to explore differences of concentration of minerals and infant/maternal characteristics across clusters. Logistic regression was used to estimate difference of probability of food group consumption across three human milk clusters.

The MetaboAnalyst platform^2^ was utilized to perform metabolic pathway enrichment analysis and pathway topology analysis of differential metabolites based on KEGG database (17).

## 3 Results

### 3.1 Sample characteristics

Maternal and infant characteristics were summarized in Table 1. Overall, the present study enrolled 200 mother-infant dyads from seven cities in China, with a mean maternal age of 30.0 ± 5.4 years. The infants had a mean birth weight and body length of 3,361 g and 50.1 cm, respectively. The sample size for the second- and sixth-month lactation stages were 106 (53.0%) and 94 (47.0%), respectively.

**Table 1:** Maternal and infant characteristics^1^.

Characteristics	Total (*n* = 200)	
	Mean	SD
Maternal characteristics		
Age, y	30.0	5.4
BMI, kg/m^2^	22.8	2.8
City, *n* (%)		
Beijing	27 (13.5%)	
Chengdu	29 (14.5%)	
Guangzhou	25 (12.5%)	
Jinhua	30 (15.0%)	
Lanzhou	36 (18.0%)	
Weihai	20 (10.0%)	
Zhengzhou	33 (16.5%)	
Infant characteristics		
Sex, *n* (%)		
Male	114 (57.0%)	
Female	86 (43.0%)	
Birth weight, g	3361.0	659.1
Birth length, cm	50.1	2.4
Age, *n* (%)		
2 months	106 (53.0%)	
6 months	94 (47.0%)	
Z score		
WAZ	0.57	1.73
LAZ	0.14	2.09
WLZ	0.90	2.50

^1^WAZ, weight-for-age z score, LAZ, length-for-age z score, WLZ, weight-for-length z score.

### 3.2 Description of element concentrations

**Figure 1:**
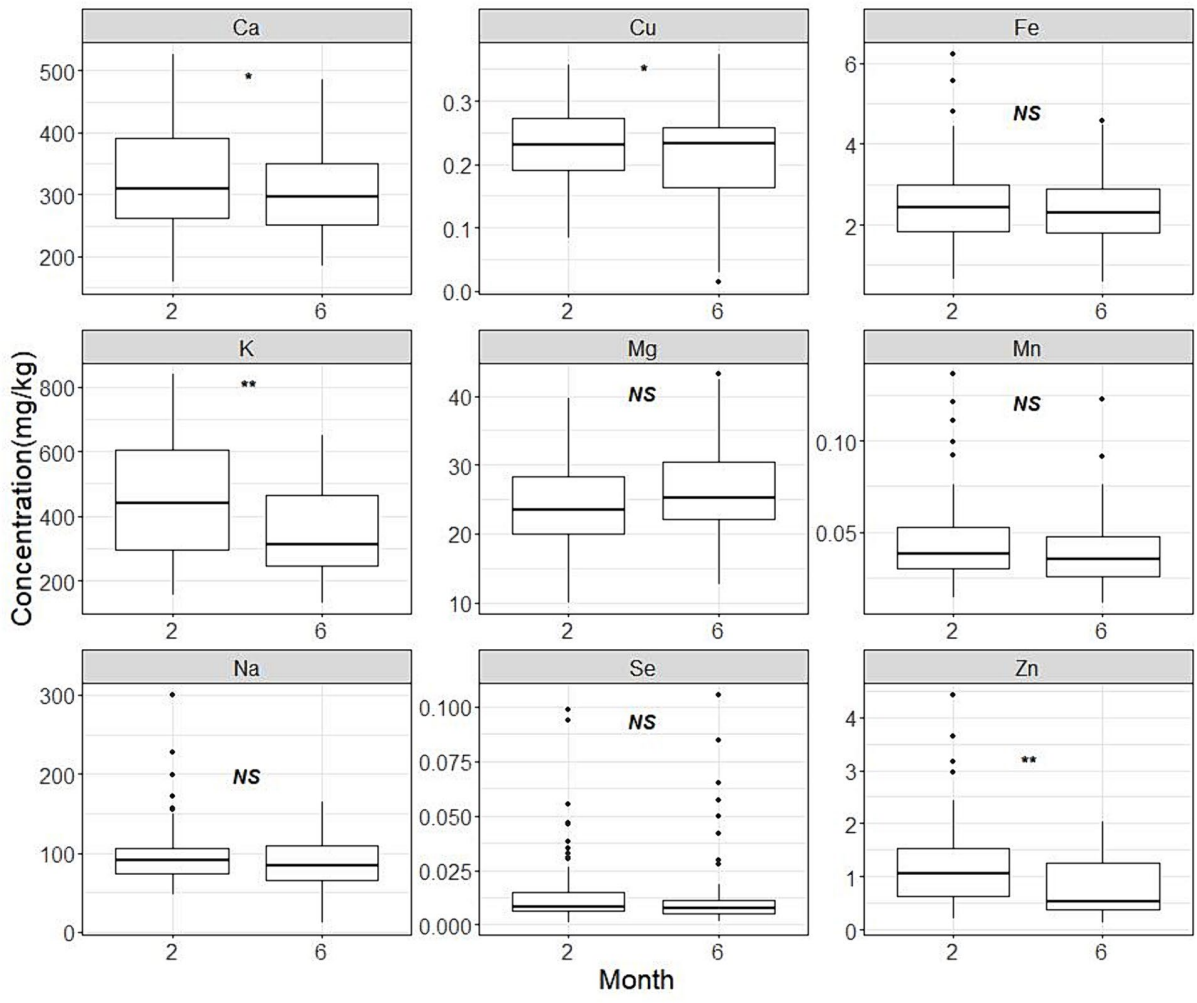
Human milk mineral contents in month 2 and month 6. The panel marked with ‘*’ or ‘**’ indicates a statistically significant difference in the concentration of this element between lactation periods at 2 months and 6 months of age. The content of potassium and zinc significantly (*p* < 0.001) dropped by 23 and 34%, respectively. Similarly, Copper and calcium demonstrated a notable declining pattern (*p* = 0.043 and 0.039, respectively).

The descriptive statistical analysis results for each element were presented in Table 2. Potassium is found to be the most abundant element on average, followed by calcium, sodium and magnesium. Figure 1 shows box plots that illustrate the distribution characteristics of each element’s contents in different lactation stages. The contents of most mineral and trace elements (except for magnesium) showed tendencies to decrease during the established mature lactation stages. Particularly, the content of potassium and zinc significantly (*p* < 0.001) dropped by 23 and 34%, respectively. While copper and calcium also displayed a significant decreasing trend (*p* = 0.043 and 0.039, respectively).

**Table 2:** Concentrations of human milk minerals and trace elements (mg/kg).

Element	Mean	SD	1st Quartile	Median	3rd Quartile
Na	91.681	34.946	69.030	89.605	106.320
K	408.629	168.566	271.600	375.303	547.200
Cu	0.221	0.067	0.180	0.232	0.265
Mg	25.045	6.492	20.550	24.332	29.558
Fe	2.427	0.899	1.827	2.366	2.949
Zn	0.987	0.680	0.443	0.796	1.453
Mn	0.041	0.020	0.028	0.036	0.051
Ca	316.361	75.498	256.500	301.907	370.100
Se	0.013	0.016	0.006	0.008	0.013

### 3.3 Cluster analysis

Characteristics of mineral clusters is displayed in Table 3. Eight out of the 24 indices suggested that the optimal number of clusters was three, with sample sizes of 96, 18 and 86, respectively. The mean contents of each element in these clusters were extremely different (*p*<0.001). Cluster I was characterized as the highest levels of potassium, magnesium and calcium, while the lowest levels of copper, zinc, manganese and selenium. Cluster II showed the most abundant sodium, iron, zinc, manganese and selenium. In contrast, human milk samples of Cluster III had the lowest levels of Na, K, Mg, Fe and Ca.

**Table 3:** Characteristics of human milk mineral clusters^1^.

Element	Cluster I *N* = 96 (mg/Kg)		Cluster II *N* = 18 (mg/Kg)		Cluster III *N* = 86 (mg/Kg)		*p* ^2^
	Mean	SD	Mean	SD	Mean	SD	
Na	97.02	30.77	120.84	63.09	79.62	25.40	<0.01
K	510.06	145.56	500.79	138.50	276.11	88.42	<0.01
Cu	0.18	0.06	0.25	0.07	0.26	0.04	<0.01
Mg	27.25	6.36	24.54	6.30	22.69	5.86	<0.01
Fe	2.65	0.82	3.13	1.36	2.02	0.66	<0.01
Zn	0.61	0.35	1.93	1.04	1.22	0.57	<0.01
Ca	350.00	77.41	313.72	98.19	279.37	45.33	<0.01
Mn	0.035	0.013	0.074	0.034	0.041	0.016	<0.01
Se	0.00	0.006	0.048	0.030	0.010	0.008	<0.01

^1^The mineral clusters were calculated by NbClust R package (version 3.0.1.) (16) based on K-means clustering algorithm.

^2^The significant *p*-values across clusters were calculated by multiple linear regressions, which were adjusted for infant age, infant sex and city. *p*<0.05 means statistically significant.

### 3.4 Differences of infant anthropometries and maternal BMI

The results of multiple linear regression on infant and maternal physical characteristics across clusters are presented in Table 4. The results suggest that infants in cluster I have higher scores in terms of body length, body weight, WAZ, LAZ and HCZ when compared to those in the other two clusters. The body Weight of infants was significantly different across clusters under the raw model (*p*<0.01), and these differences remained statistically significant even after adjusting for city, infant age and infant sex (*p* = 0.04). The body length and LAZ in the raw model showed significant differences across clusters (*p* = 0.02 and *p* = 0.03), but significant differences were not observed after adjusting for city and infant month factors. However, WAZ exhibited significant differences among clusters in the adjusted model (*p* = 0.02). There are no significant differences in maternal BMI across three clusters.

**Table 4:** Differences of maternal and infant characteristics across clusters of human milk minerals.

Characteristic	Cluster I	Cluster II	Cluster III	*p* ^4^	*p* ^lm5^
	Mean ± SD	Mean ± SD	Mean ± SD		
Body Length (cm)	61.94 ± 7.08	56.80 ± 6.07	60.43 ± 6.82	0.02	0.27
Body Weight (kg)	6.77 ± 2.04	5.11 ± 1.40	6.57 ± 1.93	<0.01	0.04
WAZ^1^	0.74 ± 1.64	−0.25 ± 1.50	0.44 ± 1.86	0.06	0.02
LAZ^2^	0.60 ± 2.03	−0.34 ± 1.88	−0.22 ± 2.12	0.03	0.25
HCZ^3^	0.66 ± 1.43	−0.04 ± 1.77	0.31 ± 1.74	0.16	0.45
Maternal BMI	23.05 ± 3.00	23.46 ± 2.71	22.52 ± 2.65	0.30	0.39

^1^WAZ, weight for age Z score.

^2^LAZ, Length for age Z score.

^3^HCZ, weight-for-head circumference z scores.

^4^*p* means the p-values calculated by multiple linear regressions for raw model (no adjustment applied), *p*<0.05 means statistically significant.

^5^*p*^lm^ means the *p*-values obtained from multiple linear regressions for model 1, which has been adjusted for city, infant age, and infant sex, *p*<0.05 means statistically significant.

### 3.5 Food group consumption across three clusters

Food group consumption across the three human milk mineral clusters is provided in Supplementary Table S5. The consumption of both white and yellow tubers, coarse grains, meat, and milk varied significantly across the three clusters (*p* < 0.05). Cluster I showed a higher consumption rate of white and yellow tubers, dark vegetables, beans, meat, and milk compared to Cluster II. In contrast, Cluster III had a lower intake rate of these foods than Cluster I. The probability of consumption for eight food categories across three clusters of human milk minerals is available as Supplementary Table S6. In model 1, there were associations observed between several food categories and clusters. However, in model 2, after adjusted for city and lactation stage, the correlation was found to be non-significant. Maternal daily mineral supplements intakes across clusters were analyzed (Supplementary Table S7). There are no significant differences in mineral supplement consumption across the three clusters.

### 3.6 Metabolomic differences across clusters

**Figure 2:**
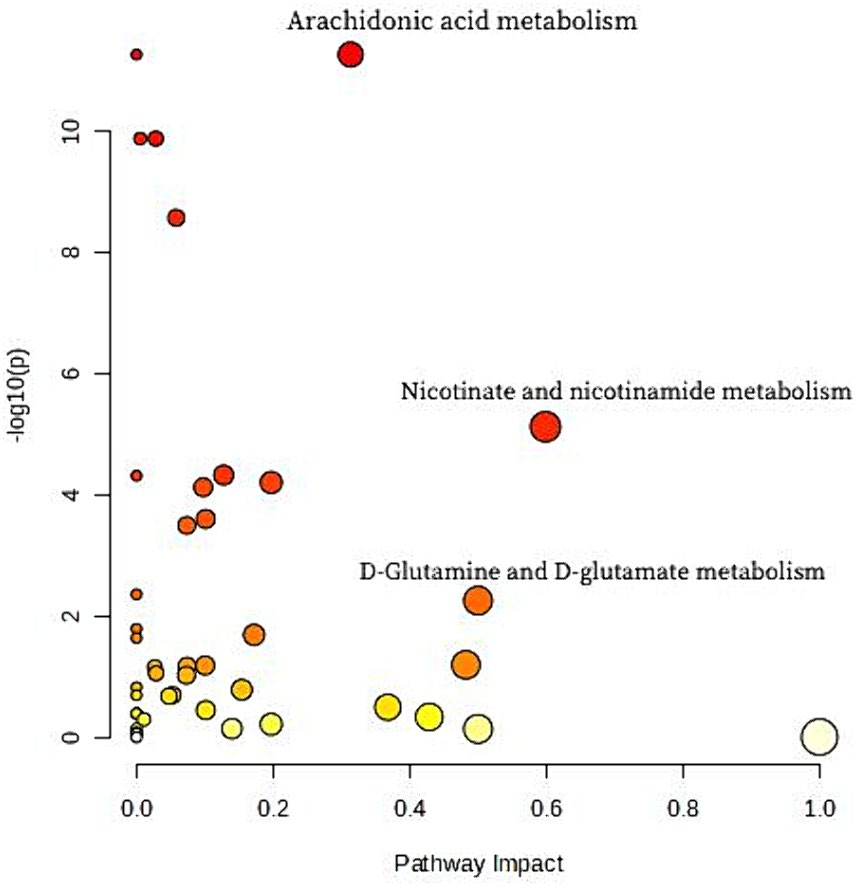
The entire set of matched differential metabolic pathways between human milk of Cluster I and other clusters based on *p* values from pathway enrichment analysis and pathway impact values derived from pathway topology analysis. Each node represents a distinct metabolism pathway. It was observed that Cluster I and the other two clusters exhibited significant differences in multiple metabolic pathways. The Arachidonic acid metabolism pathway, with a most significant *p* value is the major distinguishing feature of Cluster I from the other two clusters. Additionally, Nicotinate and nicotinamide metabolism pathway and D-Glutamine and D-glutamate metabolism pathway are also differential metabolic pathways that have a remarkable impact on Cluster I compared to the other two clusters.

Metabolic pathway analysis was carried out to explore potential metabolic differences between human milk of Cluster I and of other clusters. Figure 2 presents the entire set of matched pathways based on *p* values from pathway enrichment analysis and pathway impact values derived from pathway topology analysis. It was observed that Cluster I and the other two clusters exhibited significant differences in multiple metabolic pathways including arachidonic acid pathway, Nicotinate and nicotinamide metabolism pathway and D-Glutamine and D-glutamate metabolism pathway. Nine metabolites involved in the three differential metabolic pathways are listed in Table 5. Difference of metabolite abundance across three human milk mineral cluster are listed in Supplementary Table S8.

**Table 5:** Metabolites of the three differential metabolic pathways.

Metabolites	Cluster 1		Cluster 2 and 3		*p*	FDR	Fold change
	Mean	SD	Mean	SD			
Arachidonic acid	1.30E-05	3.39E-05	3.26E-05	2.63E-05	4.98E-12	6.18E-10	0.40
Glutamine	1.44E-02	5.61E-03	1.10E-02	6.02E-03	0.004	0.025	1.31
L-Glutamic acid	1.33E-03	2.45E-04	1.36E-03	3.02E-04	0.789	0.906	0.98
Pyroglutamic acid	6.01E-05	3.35E-05	3.76E-05	3.39E-05	0.011	0.052	1.60
Niacinamide	2.20E-03	1.53E-03	3.47E-03	2.45E-03	1.44E-06	2.10E-05	0.64
Nicotinic acid	3.49E-05	7.12E-06	5.61E-05	1.24E-04	0.032	0.113	0.62
Nicotinamide ribotide	4.51E-05	4.43E-05	6.80E-05	7.24E-05	0.042	0.131	0.66
NAD	5.93E-06	3.75E-06	5.46E-06	3.81E-06	0.053	0.159	1.09
1-Methylnicotinamide	2.10E-04	7.26E-05	2.21E-04	8.33E-05	0.333	0.548	0.95

## 4 Discussion

Minerals are indispensable nutrients for the human body, which play essential roles in the process of promoting and sustaining bone growth (1) Our results indicate that specific human milk mineral pattern (highest levels of potassium, magnesium and calcium, while the lowest levels of copper, zinc, manganese and selenium) is associated with greater infant growth, which seems to be related to altered metabolic signature of human milk.

The levels of macro minerals, including sodium, magnesium, potassium, and calcium in this study are comparable to those reported by Sun Zhongqing et al., (4) for Chinese mature human milk using the same detection method. Specifically, the P50 percentiles were found to be 109.2 mg/kg for sodium, 471.2 mg/kg for potassium, 253.3 mg/kg for calcium, and 27.3 mg/kg for magnesium. The zinc levels were slightly lower compared to the findings reported by Liu Qiang et al. (18) and Zhao et al. (5) during the 1–6 months postpartum period, which indicated mean levels of approximately 1.5 mg/kg. The concentration of iron in this study is higher than that reported by other researchers in China (5, 19, 20) and Japan, with an average content of iron during the 6 months postpartum ranging from 0.9 to 1.63 mg/kg, using the same detection method. The concentrations of copper and selenium were comparable to those measured by Liu Liping using ICP-MS (19) and other similar studies on Chinese human milk (3). A study conducted in seven cities across China revealed that the concentration of Manganese in breast milk was significantly higher in Harbin and Hohhot compared to other regions. The mean value obtained from our study approximated the levels observed in these two northern Chinese cities (21). Several previous studies from other countries also reported a downtrend in the mineral and trace element concentrations including copper, zinc, calcium, iron, potassium, sodium, and selenium (7, 22).

Meat is a rich source of heme iron, which is absorbed much more easily by the human body compared to non-heme iron found in plant-based foods. Milk is well-known for its high calcium content. Our study found that an increase in meat and milk consumption by mothers can improve the infants’ body length, and weight (as shown in Table 4). This highlights the importance of lactating mothers to maintain a nutrient-dense diet to ensure infant growth and development. It is documented that there is no significant association between maternal intake of iron and calcium with their levels in human milk (5, 23). Women who are in the same stage of lactating are likely to have comparable levels of calcium in their milk, and daily supplementation did not increase the milk level since serum calcium is normally tightly controlled by homeostatic mechanisms. Our study showed a significantly higher level of calcium in Cluster I, where the maternal dietary consumption rate of milk is the highest. However, no comparable pattern was seen when Cluster II and III were compared, and the lack of precise dietary intake information from participants is hindering the understanding of the underlying reasons.

To our knowledge, the present study is the first of its kind to explore associations between human milk mineral patterns, infant growth rate and human milk metabolomics. Arachidonic acid metabolic pathway displayed the most pronounced variation between Cluster I and the other two clusters, with the abundance of ARA being significantly lower in Cluster I. It has been reported previously that Mg^2+^ deficiency decrease conversion of free linoleic acid (LA) to ARA by impairing Δ-6 desaturase activity, therefore may lead to accumulation of free LA and its metabolites (24, 25). Interestingly, it has been shown that depletion of intracellular Mg^2+^ may reduce incorporation of exogenous ARA into tissue phospholipids (PL) by reducing synthesis of arachidonoyl-coenzyme A (26). Moreover, bindings of Mg^2+^ to protein kinase C through its specific binding site may reduce the activity of this enzyme, which may in turn elevate concentration of free ARA and its metabolites (27, 28). Similarly, zinc has been reported to be positively associated with plasma and liver phospholipids ARA levels by altering Δ-5 and Δ-6 desaturase activity (29, 30). Collectively, all these are in line with our results that Cluster I with the highest Mg while the lowest Zn showed decreased free ARA abundance.

Studies have demonstrated that ARA plays an important role in regulating bone formation, mineral metabolism, and balance during the growth and development of infants. Firstly, ARA serves as a precursor for the synthesis of prostaglandin E2 (PGE2), a potent bone-resorbing agent that stimulates the production of insulin-like growth factor 1 (IGF-1) and insulin-like growth factor binding protein 5 (IGFBP-5). These factors effectively promote osteoblast growth, particularly in long bones (31, 32). Furthermore, ARA and PGE2 are closely linked to signal pathway of 1a,25-(OH)_2_D_3_ which promotes active calcium absorption. Moreover, prostaglandin can modulate the activity of 1a,25-(OH)_2_D_3_ and influence the protein kinase C (PKC) signaling pathway through differential regulation of 1a,25-(OH)_2_D_3_ and 24R,25-(OH)_2_D_3_ in growth plate chondrocytes. Arachidonic acid induces an increase in PKC activity in growth zone cells while decreasing it in resting zone cells, thereby promoting proliferation and calcification of chondrocytes within the epiphyseal growth plate (33–35).Thirdly, Arachidonic acid regulates the absorption and remodeling of bone tissue, thereby regulating the adaptability of bone tissue to physiological stress (36).Therefore, the differential impact of Arachidonic acid metabolic pathway on cluster I is a contributing factors in promoting growth of osteoblasts and growth plate chondrocytes which play crucial role in proper endochondral bones development. In cluster I, free ARA seems to be metabolized in greater degrees which undergoes conversion into downstream products, resulting in a lower residual ARA content. It has been reported that adequate Zn status is necessary for omega-6 fatty acid metabolism. Both FADS1 and FADS2 are Zn-dependent enzymes that are responsible for metabolizing linoleic acid to ARA. It has been reported that there is a direct link between low dietary Zn intake and delta 5 desaturase and delta 6 desaturase activities (37). It is therefore also possible that a lower Zn level of Cluster 1 may decrease FADS1 and FADS2 activities which leads to lower ARA.

Nicotinic acid and nicotinamide act as precursors in the biosynthesis of nicotinamide adenine dinucleotide (NAD), an essential cofactor that plays a pivotal role in diverse metabolic processes, including enzymatic redox reactions and energy production (38). NAD is synthesized from dietary precursors via several pathways: the *de novo* pathway, Preiss–Handler and salvage pathways which produces NAD from tryptophan, nicotinic acid (NA) and niacinamide (NAM), respectively. It has been reported that maternal nicotinamide riboside supplementation during lactation promoted growth of offspring (39). In addition, decreased NAD+ levels seem to be related to reduced bone formation (39, 40). More importantly, a recent study showed that NAD synthesized from the salvage pathway is critical for proper bone development in mice (41). In our study, human milk of cluster I showed altered signature of nicotinic acid and nicotinamide metabolism, with level of NAM, NA and nicotinamide ribotide (NMN) being significantly (*p* < 0.05, FDR ≤ 0.131) reduced while NAD (*p* = 0.053, FDR = 0.159) being elevated in cluster I. Therefore, it is likely that the increased growth rate of infant of cluster I is partly related to the altered Nicotinic acid and nicotinamide metabolism in the human milk.

Glutamine and glutamate are crucial components in maintaining normal placental function and promoting fetal development. Compelling evidence suggests that the amino acid transport system A and L, which include glutamine as a constituent, are less active in the fetuses with growth restriction (42). Study conducted by Wei Zhang et al. utilized PCA analysis to characterize the metabotypes of human milk and revealed a negative correlation between PC 1 and glutamate as well as D-glutamine metabolism. Additionally, higher scores on PC 1 were found to be associated with lower infant WAZ and LAZ (12).

In the present study, cluster I was characterized as the highest levels of K, Mg and Ca, while the lowest levels of Cu, Zn, Mn and Se. Similar distribution pattern has been reported for human milk Zn and Cu, where majority of these two elements are bounded to serum albumin (43, 44). Interestingly, considerable amount of human milk Fe and Mn are reported to be bound to lactoferrin. In contrast, nearly 60% of human milk Mg is found in low-molecular weight faction (such as citrate and phosphate) rather than protein faction, which is also true for human milk Ca (around 40%). Therefore, it seems reasonable to speculate that the unique profile of cluster I is interrelated to other components including lactoferrin, serum albumin and low-molecular weight faction. Future study is needed to elucidate mechanism of this dynamic interplay as well as its health effects.

Recently, a systematic review explored association between human milk mineral content and infant growth, with inconclusive results being reported (9). The author concluded that “high-quality research employing chronobiology and systems biology approaches is required to understand how HM components work independently and together to influence infant growth.” To our knowledge, only one study applied PCA to identify minerals patterns in lactating Guatemalan women and examined the association between mineral patterns and infant anthropometric outcomes. The results showed that a human milk mineral concentration pattern composed of higher Cu, K, Na, Se and Zn was negatively associated with WAZ and LAZ during early lactation (18–46d) (8). This is in agreement with our results that cluster I lower in Zn, Cu, Se showed significantly higher LAZ, suggesting advantages of multivariate analysis due to its ability to capture underlying interplay of high dimensional data. In addition to human milk minerals, our study further included human milk metabolomic profile, which we believe is the major strength of the present study. It has been widely documented that a large proportion of human milk minerals (such as Mg and Zn) are bounded to citrate, which may be easily freed as ions therefore may be involved in the overall metabolism (43, 44). Another systematic review conducted by Reyes et al. (9) investigated the relationship between HM Zn and infant weight and length-related outcomes, with generally varying results. The reason for the varying results across studies remains unclear but could be linked to the overall maternal nutritional status since the maternal diet influences HM protein concentrations (45), and higher HM protein concentration improves infant Zn absorption (46). Thus, improving the maternal diet would increase HM protein concentrations, leading to improved HM Zn absorption and improved infant growth outcomes. The etiology of growth retardation is likely multifactorial and affected by deficits of energy or several other limiting nutrients such as protein, iron, and vitamin A, D, and C, apart from low Zn intakes. The relationship between HM nutrients and infants’ growth is challenging to determine due to the multitude of factors that influence infant nutritional status and the bioactivity of nutrients, including genetics, health status, and other environmental factors.

The limitation of our study is the cross-sectional study design which only reveal correlation rather than causality. Another limitation is that total mineral contents were detected in our study, which is not able to reveal distribution of minerals in different human milk fraction.

To conclude, the specific mineral composition of human milk (highest levels of potassium, magnesium and calcium, while low levels of copper, zinc, manganese and selenium) is associated with increased infant growth. This may be due to alterations in the metabolic signature of human milk. Future study is needed to elucidate mechanism of dynamic interplays between human milk minerals from different fractions and specific metabolic pathways as well as their health effects.

## Data availability statement

The original contributions presented in the study are included in the article/Supplementary material, further inquiries can be directed to the corresponding authors.

## Ethics statement

The studies involving humans were approved by Shanghai Nutrition Society Ethic Committee. The studies were conducted in accordance with the local legislation and institutional requirements. Written informed consent for participation in this study was provided by the participants’ legal guardians/next of kin.

## Author contributions

HS: Formal analysis, Methodology, Software, Validation, Visualization, Writing – original draft. QX: Investigation, Validation, Writing – review & editing. YZ: Conceptualization, Investigation, Methodology, Writing – review & editing. YL: Investigation, Writing – review & editing. JP: Resources, Writing – review & editing. YX: Conceptualization, Supervision, Writing – review & editing. SJ: Conceptualization, Funding acquisition, Investigation, Methodology, Project administration, Validation, Writing – review & editing. KL: Conceptualization, Funding acquisition, Investigation, Methodology, Supervision, Validation, Writing – review & editing.

## Footnotes

^1^ http://cran.r-project.org/

^2^ www.Metaboanalyst.ca
