# Supplementary material for: An investigation of association between human milk mineral patterns and infant growth
Source: Front Nutr. 2024 Jun 19;11:1387956. doi: 10.3389/fnut.2024.1387956 (PMC11220249; doi:10.3389/fnut.2024.1387956)
Supplement: Supplementary file 1 [file Data_Sheet_1.docx]

Supplementary Material

Table S1 Main operating parameters of the instrument

| Operating parameters | Setpoint | Operating parameters | Setpoint |
| --- | --- | --- | --- |
| RF power (W) | 1200 | Scan mode | Peak hopping |
| Atomizer pressure (bar) | 0.9～1.0 | Number of sweeps | 60 |
| Auxiliary gas flow | 0.70 | Integration time (ms) | 10 |
| Plasma gas flow rate | 13.0 | Measurement points per mass | 3 |
| Sampling cone | 1.0mm (Nickel) | Measurement method | Pulse/Analog |
| Interception cone | 0.7mm (Nickel) | Peristaltic pump speed (rpm) | Analysis:30：  Rinse: 70 |

Table S2 Detection and quantification limits (LOD and LOQ) of the elements analysed

| Element | LOD  （mg/L） | LOQ  （mg/L） | Calibration range  Ug/mL | Correlation coefficient |
| --- | --- | --- | --- | --- |
| Sodium | 0.3 | 1 | 0.1-20 | 0.9997 |
| Potassium | 0.3 | 1 | 0.1-20 | 0.9997 |
| Calcium | 0.3 | 1 | 0.1-20 | 0.9997 |
| Magnesium | 0.3 | 1 | 0.1-20 | 0.9998 |
| Copper | 0.02 | 0.05 | 0.01-0.5 | 0.9999 |
| Zinc | 0.2 | 0.5 | 0.01-0.5 | 1.0000 |
| Iron | 0.3 | 1 | 0.01-0.5 | 0.9997 |
| Manganese | 0.03 | 0.1 | 0.01-0.5 | 1.0000 |
| Selenium | 0.003 | 0.01 | 1-100 ng/mL | 0.9997 |

Table S3 Food Frequency Questionnaire_Record of Household oil and salt Consumption

Interview Date: Year _ _Month _ _Day M20

| Condiment | Consumption per month (grams) |
| --- | --- |
| M21 Total Edible oil |  |
| M22 Lard |  |
| M23 Iodised salt |  |
| M24 Which vegetable oil have you consumed the most in the previous month? (single choice) | ①peanut oil ②soybean oil ③corn oil ④rapeseed oil ⑤blend oil ⑥sunflower oil ⑦olive oil ⑧sesame oil ⑨others |

Table S4. Food Frequency Questionnaire (for all age 12 and older)

***Please recall whether you ate these food items in the past week or not, and estimate the frequency and average edible amount.**

| Food Name | | **Eat/drink or not (a)**  **1 Yes**  **0 No** | **Frequency (answer only one of these) (b)** | | | **Average**  **amount for**  **each time**  **(grams or ml)** |
| --- | --- | --- | --- | --- | --- | --- |
|  |  |  | **Times**  **per**  **day** | **Times**  **per**  **week** | **Times**  **per**  **month** |  |
| **Staple food** | | | | | | |
| M25 | Rice (cooked rice/rice flour) |  |  |  |  | gram |
| M26 | Wheat products (white bread, steamed bread/noodles/dumplings), non-fried, excluding instant noodles |  |  |  |  | gram |
| M27 | Coarse grains (corn, millet, sorghum, barley, rye) |  |  |  |  | gram |
| M28 | White tuber (Potato/taro /yam) |  |  |  |  | gram |
| M29 | Yellow tuber (Sweet potatoes) |  |  |  |  | gram |
| **Beans** | | | | | | |
| M30 | Dried soybean (yellow, green or black) |  |  |  |  | gram |
| M31 | All other beans (mung bean /red  bean/kidney bean) |  |  |  |  | gram |
| M32 | Soybean milk |  |  |  |  | ml |
| M33 | Soybean powder |  |  |  |  | gram |
| M34 | Tofu, soybean milk films，bean products |  |  |  |  | gram |
| **Vegetables** | | | | | | |
| M35 | Yellow vegetables (carrots, squash, daylily and other yellow vegetables) |  |  |  |  | gram |
| M36 | Green leafy `spinach/rape vegetable/water spinach/bok choy) |  |  |  |  | gram |
| M37 | Cruciferous vegetables (Broccoli,  cauliflower) |  |  |  |  | gram |
| M38 | Other vegetables (cucumner, winter melon, Chinese cabbage eggplant and other leafy vegetables celery/lettuce, etc.) |  |  |  |  | gram |
| M39 | Fungi (mushrooms) |  |  |  |  | gram |
| **Fruits** | | | | | | |
| M40 | Yellow fruit (such as mango, orange, orange, papaya, cantaloupe, apricot, pineapple, pipa, persimmon, sea buckthorn fruit, etc.) |  |  |  |  | gram |
| M41 | Watermelon/honeydew melon/other melons |  |  |  |  | gram |
| M42 | All other fresh (apples, berries, bananas, kiwi, etc.) |  |  |  |  | gram |
| **Meat** | | | | | | |
| M43 | Chicken, duck, goose, pigeon, quail  (fried/non-fried) |  |  |  |  | gram |
| M44 | Pork/Beef/ Lamb/mutton/other non-processed meats |  |  |  |  | gram |
| M45 | Avian and livestock viscera |  |  |  |  | gram |
|  | Aquatic products (fish,shellfish and their viscera, including algae) |  |  |  |  | gram |
| **Milk** | | | | | | |
|  | Milk and milk products (measured in liquids) |  |  |  |  | ml |
| **Eggs** | | | | | | |
| M46 | Fresh eggs(chicken/duck/quail egg) |  |  |  |  | gram |
|  |  |  |  |  |  |  |
| M47 | preserved eggs |  |  |  |  | gram |
| **Snacks** | | | | | | |
| M48 | Nuts (peanut/ sun flower seeds/ pumpkin seeds/watermelon seeds/other seeds) |  |  |  |  | gram |
| **Nutritional Supplements** | | | | | | |
| M49 | Nutritional supplement 1 |  |  |  |  | gram |
| M50 | Nutritional supplement 2 |  |  |  |  | gram |
| M51 | Nutritional supplement 3 |  |  |  |  | gram |

* Dairy products are calculated based on fresh milk, while powdered milk is diluted at a ratio of 1 to 6.

Cheese is multiplied by 10 to calculate its weight as fresh milk.

Common nutritional supplements: protein powder, calcium tablets, iron, zinc, DHA (fish oil, etc.).

Table S5 Food group consumption across the three human milk mineral clusters

| Characteristic | Cluster I | Cluster II | Cluster III | *P* |
| --- | --- | --- | --- | --- |
|  | Mean±SD | Mean±SD | Mean±SD |  |
| Coarse grain | 93.75% | 82.35% | 79.76% | 0.02 |
| White tuber | 75.79% | 43.75% | 65.48% | 0.03 |
| Yellow tuber | 55.79% | 0.00% | 35.29% | ＜0.001 |
| Dark vegetable | 97.92% | 93.75% | 96.47% | 0.63 |
| Yellow fruit | 82.11% | 68.75% | 68.60% | 0.09 |
| Other vegetables | 96.88% | 93.75% | 95.29% | 0.78 |
| Bean | 87.50% | 75.00% | 77.65% | 0.16 |
| Nut | 64.21% | 37.50% | 52.94% | 0.08 |
| Egg | 94.74% | 94.12% | 90.59% | 0.55 |
| Meat | 95.83% | 82.35% | 84.52% | 0.02 |
| Milk | 84.21% | 50.00% | 77.91% | 0.01 |
| Fish & aquatic products | 80.21% | 68.75% | 70.37% | 0.27 |
| Tuber(white & yellow tuber) | 77.89% | 43.75% | 67.06% | 0.01 |

Table S6 Differences of probability of food group consumption across three human milk mineral clusters.

|  |  | Cluster 2 | | | Cluster 3 | | |
| --- | --- | --- | --- | --- | --- | --- | --- |
|  |  | OR | 95% CI | | OR | 95% CI | |
| Coarse grain | Model 1 | 0.35 | 0.08 | 1.56 | 0.25 | 0.09 | 0.67 |
|  | Model 2 | 0.63 | 0.09 | 4.34 | 0.40 | 0.07 | 2.33 |
| Starchy roots | Model 1 | 0.25 | 0.08 | 0.76 | 0.55 | 0.28 | 1.08 |
|  | Model 2 | 0.41 | 0.09 | 1.92 | 0.85 | 0.19 | 3.70 |
| Fruits | Model 1 | 0.60 | 0.18 | 2.01 | 0.43 | 0.21 | 0.88 |
|  | Model 2 | 1.10 | 0.22 | 5.58 | 1.13 | 0.27 | 4.66 |
| Meat and meat products | Model 1 | 0.22 | 0.04 | 1.09 | 0.23 | 0.07 | 0.74 |
|  | Model 2 | 0.11 | 0.01 | 1.73 | 0.08 | 0.01 | 1.04 |
| Soybean and soybean products | Model 1 | 0.37 | 0.10 | 1.36 | 0.51 | 0.23 | 1.14 |
|  | Model 2 | 1.45 | 0.21 | 10.04 | 2.84 | 0.49 | 16.39 |
| Nuts | Model 1 | 0.34 | 0.11 | 1.02 | 0.63 | 0.34 | 1.14 |
|  | Model 2 | 0.82 | 0.20 | 3.34 | 1.32 | 0.41 | 4.25 |
| Dairy products | Model 1 | 0.19 | 0.06 | 0.60 | 0.66 | 0.31 | 1.40 |
|  | Model 2 | 0.32 | 0.07 | 1.47 | 1.21 | 0.30 | 4.93 |
| Fish and aquatic products | Model 1 | 0.62 | 0.19 | 2.04 | 0.55 | 0.27 | 1.12 |
|  | Model 2 | 0.53 | 0.11 | 2.46 | 0.28 | 0.08 | 0.999 |

Model 1: Logistic regression adjusted for lactation stage.

Model 2: Logistic regression adjusted for city and lactation stage.

Table S7 Maternal daily mineral supplement intakes across clusters

|  | Supplement consumers | Non-supplement consumers | P value |
| --- | --- | --- | --- |
| Cluster 1 | 58 (60.4%) | 38 (39.6%) | 0.798 |
| Cluster 2 | 11 (61.1%) | 7 (38.9%) |  |
| Cluster 3 | 48 (55.8%) | 38 (44.2%) |  |

_ _ _ _ _ _

Table S8 Difference of metabolite abundance across three human milk mineral cluster

| Metabolites | Cluster 1 | | Cluster 2 and 3 | | P | FDR | Fold change |
| --- | --- | --- | --- | --- | --- | --- | --- |
|  | Mean | SD | Mean | SD |  |  |  |
| Delta-12-Prostaglandin J2 | 7.05E-06 | 2.94E-05 | 1.55E-05 | 1.72E-05 | 1.28E-15 | 3.18E-13 | 0.45 |
| Arachidonic acid | 1.30E-05 | 3.39E-05 | 3.26E-05 | 2.63E-05 | 4.98E-12 | 6.18E-10 | 0.40 |
| 13S-hydroxyoctadecadienoic acid | 3.91E-06 | 6.26E-06 | 8.64E-06 | 6.96E-06 | 1.12E-11 | 7.66E-10 | 0.45 |
| 4,8 Dimethylnonanoyl carnitine | 2.70E-05 | 1.06E-04 | 4.13E-05 | 4.62E-05 | 1.24E-11 | 7.66E-10 | 0.66 |
| Dihydro-6-isopropyl-2,4-dimethyl-4H-1,3,5-dithiazine | 1.42E-05 | 2.50E-05 | 3.73E-05 | 3.29E-05 | 1.98E-11 | 9.82E-10 | 0.38 |
| Cholesterol | 2.39E-05 | 2.72E-05 | 7.34E-05 | 9.34E-05 | 1.38E-10 | 5.27E-09 | 0.33 |
| 10-Oxo-11-octadecen-13-olide | 1.31E-05 | 2.15E-05 | 3.06E-05 | 3.95E-05 | 1.49E-10 | 5.27E-09 | 0.43 |
| DG(22:5(7Z,10Z,13Z,16Z,19Z)/16:1(9Z)/0:0) | 8.33E-06 | 7.73E-06 | 1.95E-05 | 1.37E-05 | 2.83E-10 | 8.76E-09 | 0.43 |
| Nandrolone | 2.23E-05 | 9.21E-05 | 3.68E-05 | 4.52E-05 | 1.84E-09 | 5.08E-08 | 0.61 |
| N2-gamma-Glutamylglutamine | 1.06E-04 | 8.08E-05 | 5.61E-05 | 8.42E-05 | 6.38E-09 | 1.58E-07 | 1.89 |
| Alpha-dimorphecolic acid | 3.04E-04 | 6.05E-04 | 7.33E-04 | 6.18E-04 | 9.39E-09 | 2.12E-07 | 0.42 |
| Styrene | 5.96E-05 | 5.16E-05 | 8.04E-05 | 3.31E-05 | 4.22E-08 | 8.71E-07 | 0.74 |
| 5'-Methylthioadenosine | 1.12E-03 | 3.86E-04 | 7.87E-04 | 3.95E-04 | 1.21E-07 | 2.32E-06 | 1.42 |
| Dimethyl dialkyl ammonium chloride | 1.81E-06 | 5.62E-06 | 9.05E-05 | 3.19E-04 | 5.46E-07 | 9.67E-06 | 0.02 |
| Biliverdin | 6.87E-05 | 4.89E-05 | 3.75E-05 | 6.78E-05 | 1.13E-06 | 1.86E-05 | 1.83 |
| Furcelleran | 5.67E-06 | 4.65E-06 | 2.85E-06 | 3.50E-06 | 1.27E-06 | 1.97E-05 | 1.99 |
| **Niacinamide** | **2.20E-03** | **1.53E-03** | **3.47E-03** | **2.45E-03** | **1.44E-06** | **2.10E-05** | **0.64** |
| 1-Methyl-1,3-cyclohexadiene | 1.70E-04 | 9.16E-05 | 2.24E-04 | 8.92E-05 | 1.84E-06 | 2.54E-05 | 0.76 |
| (9S,10E,12Z,15Z)-9-Hydroxy-10,12,15-octadecatrienoic acid | 4.08E-05 | 1.10E-04 | 5.76E-05 | 5.07E-05 | 2.62E-06 | 3.41E-05 | 0.71 |
| Epsilon-caprolactam | 4.36E-05 | 2.48E-05 | 6.48E-05 | 4.25E-05 | 5.62E-06 | 6.97E-05 | 0.67 |
| o-Xylene | 4.88E-05 | 3.82E-05 | 6.22E-05 | 2.73E-05 | 1.47E-05 | 1.74E-04 | 0.78 |
| LysoPE(0:0/18:3(6Z,9Z,12Z)) | 1.65E-05 | 1.71E-05 | 9.97E-06 | 1.62E-05 | 1.84E-05 | 2.07E-04 | 1.66 |
| Guanosine | 5.58E-06 | 3.72E-06 | 3.94E-06 | 3.55E-06 | 3.35E-05 | 3.62E-04 | 1.42 |
| Xanthotoxol glucoside | 1.28E-04 | 5.26E-05 | 1.03E-04 | 7.82E-05 | 4.47E-05 | 4.61E-04 | 1.24 |
| 5,12-dihydroxy-6,8,10,14,17-eicosapentaenoic acid | 9.52E-06 | 3.01E-05 | 1.68E-05 | 1.68E-05 | 5.17E-05 | 5.13E-04 | 0.57 |
| L-Lysine | 2.55E-04 | 1.58E-04 | 3.61E-04 | 2.33E-04 | 1.40E-04 | 0.001 | 0.71 |
| N-Methylnicotinamide | 3.11E-04 | 1.22E-04 | 2.46E-04 | 1.45E-04 | 1.48E-04 | 0.001 | 1.27 |
| Cytosine | 1.68E-03 | 1.18E-03 | 1.11E-03 | 8.25E-04 | 3.65E-04 | 0.003 | 1.52 |
| Adenosine | 9.05E-04 | 6.43E-04 | 6.46E-04 | 7.24E-04 | 3.90E-04 | 0.003 | 1.40 |
| Pipecolic acid | 1.68E-04 | 7.16E-05 | 2.11E-04 | 9.76E-05 | 4.02E-04 | 0.003 | 0.80 |
| Squamolone | 4.64E-05 | 7.19E-06 | 4.26E-05 | 8.17E-06 | 4.66E-04 | 0.004 | 1.09 |
| Cytidine | 2.34E-04 | 1.91E-04 | 3.12E-04 | 1.77E-04 | 4.70E-04 | 0.004 | 0.75 |
| 4-Aminohippuric acid | 4.58E-05 | 3.31E-05 | 3.63E-05 | 1.46E-05 | 6.71E-04 | 0.005 | 1.26 |
| 1,3-Diisopropylbenzene | 5.37E-05 | 3.24E-05 | 6.61E-05 | 3.36E-05 | 0.001 | 0.008 | 0.81 |
| 2-trans-6-cis-Dodecadienal | 2.70E-05 | 1.94E-05 | 3.42E-05 | 1.92E-05 | 0.001 | 0.008 | 0.79 |
| LysoPE(16:0/0:0) | 4.58E-05 | 3.26E-05 | 6.86E-05 | 5.14E-05 | 0.001 | 0.008 | 0.67 |
| Pyrrolidonecarboxylic acid | 6.26E-04 | 1.96E-04 | 5.35E-04 | 1.83E-04 | 0.001 | 0.009 | 1.17 |
| Pyrrolidine | 5.09E-05 | 4.78E-05 | 3.46E-05 | 3.52E-05 | 0.002 | 0.012 | 1.47 |
| MG(22:2(13Z,16Z)/0:0/0:0) | 1.30E-04 | 7.12E-05 | 1.07E-04 | 6.65E-05 | 0.002 | 0.012 | 1.21 |
| N-Acetylhistidine | 3.71E-05 | 1.54E-05 | 4.36E-05 | 1.65E-05 | 0.002 | 0.015 | 0.85 |
| Prolyl-Arginine | 2.51E-06 | 4.51E-06 | 5.91E-06 | 1.03E-05 | 0.003 | 0.017 | 0.42 |
| 1H-Pyrrole-2-carboxaldehyde | 3.83E-04 | 9.25E-05 | 4.18E-04 | 9.48E-05 | 0.003 | 0.017 | 0.91 |
| Cytidine monophosphate | 1.27E-04 | 6.78E-05 | 1.00E-04 | 5.75E-05 | 0.004 | 0.023 | 1.27 |
| Glutamine | 1.44E-02 | 5.61E-03 | 1.10E-02 | 6.02E-03 | 0.004 | 0.025 | 1.31 |
| Adenine | 1.33E-04 | 7.89E-05 | 1.12E-04 | 8.93E-05 | 0.005 | 0.029 | 1.19 |
| Glycerophosphocholine | 4.59E-02 | 5.96E-03 | 4.28E-02 | 8.16E-03 | 0.005 | 0.029 | 1.07 |
| 2,4-Dimethyloxazole | 1.26E-04 | 1.78E-05 | 1.36E-04 | 3.68E-05 | 0.006 | 0.033 | 0.92 |
| 4-Aminophenol | 4.30E-04 | 1.06E-04 | 3.90E-04 | 1.11E-04 | 0.008 | 0.039 | 1.10 |
| Urocanic acid | 2.74E-04 | 2.62E-04 | 2.08E-04 | 2.39E-04 | 0.010 | 0.050 | 1.32 |
| LysoPE(20:1(11Z)/0:0) | 3.31E-05 | 2.46E-05 | 4.82E-05 | 4.04E-05 | 0.010 | 0.050 | 0.69 |
| Citrulline | 9.02E-04 | 3.31E-04 | 7.79E-04 | 3.34E-04 | 0.010 | 0.050 | 1.16 |
| Pyroglutamic acid | 6.01E-05 | 3.35E-05 | 3.76E-05 | 3.39E-05 | 0.011 | 0.052 | 1.60 |
| L-Proline | 9.22E-05 | 1.36E-05 | 8.74E-05 | 1.31E-05 | 0.012 | 0.056 | 1.05 |
| Anserine | 2.96E-05 | 4.92E-05 | 2.12E-05 | 3.57E-05 | 0.014 | 0.063 | 1.40 |
| Pivaloylcarnitine | 4.23E-04 | 3.65E-04 | 4.56E-04 | 1.24E-03 | 0.015 | 0.069 | 0.93 |
| 2-O-(6-Phospho-alpha-mannosyl)-D-glycerate | 9.14E-04 | 4.46E-04 | 1.05E-03 | 4.52E-04 | 0.016 | 0.070 | 0.87 |
| UDP-N-acetyl-alpha-D-galactosamine | 1.43E-05 | 1.18E-05 | 1.07E-05 | 8.73E-06 | 0.018 | 0.078 | 1.33 |
| Ecgonine | 1.02E-04 | 5.65E-05 | 1.26E-04 | 1.04E-04 | 0.018 | 0.078 | 0.81 |
| 1-Methylguanosine | 4.12E-04 | 9.71E-05 | 3.81E-04 | 1.19E-04 | 0.021 | 0.088 | 1.08 |
| Spinatoside | 3.21E-05 | 2.50E-05 | 2.59E-05 | 2.02E-05 | 0.022 | 0.091 | 1.24 |
| beta-Lactose | 5.23E-06 | 4.77E-06 | 7.49E-06 | 6.52E-06 | 0.022 | 0.091 | 0.70 |
| 2-Acetyl-3-ethylpyrazine | 1.10E-04 | 2.17E-05 | 1.03E-04 | 2.29E-05 | 0.023 | 0.094 | 1.06 |
| Linoleamide | 2.63E-06 | 1.12E-06 | 3.17E-06 | 2.32E-06 | 0.024 | 0.095 | 0.83 |
| 4-(3,4-Dihydroxyphenyl)-2-hydroxy-1H-phenalen-1-one | 8.77E-05 | 1.91E-05 | 9.41E-05 | 2.18E-05 | 0.027 | 0.106 | 0.93 |
| 1-Pyrroline | 2.64E-05 | 1.60E-05 | 2.18E-05 | 9.46E-06 | 0.030 | 0.113 | 1.21 |
| Polyoxyethylene (600) monoricinoleate | 2.76E-05 | 2.42E-05 | 3.56E-05 | 3.12E-05 | 0.031 | 0.113 | 0.78 |
| N4-Acetylcytidine | 1.44E-05 | 4.88E-06 | 1.34E-05 | 5.77E-06 | 0.031 | 0.113 | 1.08 |
| Quercetagetin 3'-methylether 7-glucoside | 4.62E-05 | 3.09E-05 | 3.66E-05 | 2.34E-05 | 0.032 | 0.113 | 1.26 |
| **Nicotinic acid** | **3.49E-05** | **7.12E-06** | **5.61E-05** | **1.24E-04** | **0.032** | **0.113** | **0.62** |
| Palmitoylethanolamide | 2.13E-05 | 1.34E-05 | 2.62E-05 | 2.68E-05 | 0.032 | 0.113 | 0.81 |
| 3-Acetamidobutanal | 2.09E-05 | 5.55E-06 | 2.27E-05 | 6.71E-06 | 0.033 | 0.113 | 0.92 |
| 3-Hydroxyisovalerylcarnitine | 3.97E-05 | 2.77E-05 | 3.67E-05 | 5.61E-05 | 0.033 | 0.113 | 1.08 |
| Uridine | 5.20E-05 | 2.11E-05 | 6.04E-05 | 2.66E-05 | 0.033 | 0.113 | 0.86 |
| Trigonelline | 6.08E-05 | 1.75E-04 | 1.60E-04 | 5.21E-04 | 0.035 | 0.117 | 0.38 |
| Arecaidine | 3.14E-05 | 5.84E-06 | 2.96E-05 | 5.28E-06 | 0.035 | 0.117 | 1.06 |
| 2-Methoxy-3,5-dimethylpyrimidine | 9.11E-05 | 2.99E-05 | 8.25E-05 | 2.23E-05 | 0.039 | 0.125 | 1.10 |
| N2,N2-Dimethylguanosine | 4.58E-05 | 1.38E-05 | 4.23E-05 | 1.56E-05 | 0.039 | 0.125 | 1.08 |
| L-Palmitoylcarnitine | 2.63E-06 | 3.78E-06 | 3.97E-06 | 5.81E-06 | 0.039 | 0.125 | 0.66 |
| **Nicotinamide ribotide** | **4.51E-05** | **4.43E-05** | **6.80E-05** | **7.24E-05** | **0.042** | **0.131** | **0.66** |
| Norvaline | 2.53E-05 | 5.88E-06 | 2.38E-05 | 5.57E-06 | 0.045 | 0.140 | 1.06 |
| Trimethylamine N-oxide | 1.89E-04 | 1.12E-04 | 2.41E-04 | 1.86E-04 | 0.047 | 0.144 | 0.79 |
| N-Hexadecanoylpyrrolidine | 2.92E-06 | 2.70E-06 | 3.80E-06 | 3.44E-06 | 0.048 | 0.144 | 0.77 |
| **NAD** | **5.93E-06** | **3.75E-06** | **5.46E-06** | **3.81E-06** | **0.053** | **0.159** | **1.09** |
| D-Proline | 4.75E-03 | 9.16E-04 | 4.53E-03 | 1.23E-03 | 0.055 | 0.162 | 1.05 |
| DHBOA-Glc | 5.21E-06 | 3.47E-06 | 6.08E-06 | 3.83E-06 | 0.056 | 0.165 | 0.86 |
| N-Acetyl-L-alanine | 3.17E-04 | 2.62E-04 | 3.62E-04 | 2.35E-04 | 0.057 | 0.166 | 0.87 |
| Uracil | 3.26E-04 | 1.30E-04 | 3.75E-04 | 1.58E-04 | 0.059 | 0.167 | 0.87 |
| 3,4',5,6-Tetrahydroxy-3',7-dimethoxyflavone 3-glucuronide | 7.25E-05 | 4.96E-05 | 6.36E-05 | 4.46E-05 | 0.062 | 0.174 | 1.14 |
| 3-Methylguanine | 2.34E-03 | 1.16E-03 | 2.06E-03 | 1.20E-03 | 0.064 | 0.179 | 1.13 |
| 2-O-Galloylsucrose | 1.86E-05 | 1.78E-05 | 1.49E-05 | 1.27E-05 | 0.076 | 0.209 | 1.25 |
| Sialyl-Lewis X | 9.21E-06 | 5.13E-06 | 7.61E-06 | 3.50E-06 | 0.077 | 0.209 | 1.21 |
| SM(d18:1/16:0) | 1.31E-03 | 8.14E-04 | 1.51E-03 | 8.50E-04 | 0.078 | 0.209 | 0.87 |
| N-Carboxyacetyl-D-phenylalanine | 1.51E-05 | 3.42E-06 | 1.42E-05 | 3.45E-06 | 0.083 | 0.216 | 1.06 |
| Homo-L-arginine | 4.39E-05 | 3.08E-05 | 3.62E-05 | 2.29E-05 | 0.084 | 0.216 | 1.22 |
| Oleamide | 3.54E-05 | 2.11E-05 | 4.20E-05 | 4.17E-05 | 0.084 | 0.216 | 0.84 |
| Rheidin A | 1.24E-03 | 4.89E-04 | 1.11E-03 | 3.90E-04 | 0.084 | 0.216 | 1.12 |
| Pyro-L-glutaminyl-L-glutamine | 2.58E-05 | 7.60E-06 | 2.44E-05 | 8.61E-06 | 0.085 | 0.216 | 1.06 |
| AICA-riboside | 5.63E-04 | 3.62E-04 | 5.39E-04 | 5.35E-04 | 0.088 | 0.224 | 1.04 |
| 5-Aminopentanoic acid | 1.22E-03 | 6.94E-04 | 1.11E-03 | 8.63E-04 | 0.091 | 0.228 | 1.10 |
| L-Acetylcarnitine | 1.82E-02 | 2.88E-03 | 1.76E-02 | 4.70E-03 | 0.093 | 0.230 | 1.03 |
| 1-Methylinosine | 1.00E-04 | 3.50E-05 | 9.35E-05 | 3.94E-05 | 0.095 | 0.234 | 1.07 |
| 2-Fucosyllactose | 2.20E-04 | 1.45E-04 | 1.85E-04 | 1.55E-04 | 0.096 | 0.235 | 1.19 |
| (alpha-D-mannosyl)7-beta-D-mannosyl-diacetylchitobiosyl-L-asparagine, isoform A (protein) | 1.59E-03 | 3.35E-04 | 1.52E-03 | 3.40E-04 | 0.100 | 0.240 | 1.05 |
| Acetylhydrazine | 3.55E-05 | 1.05E-05 | 3.32E-05 | 9.92E-06 | 0.110 | 0.260 | 1.07 |
| Vinylacetylglycine | 5.64E-04 | 1.34E-04 | 6.04E-04 | 1.82E-04 | 0.110 | 0.260 | 0.94 |
| Solasodine | 2.65E-06 | 5.14E-06 | 1.64E-06 | 3.56E-06 | 0.113 | 0.265 | 1.62 |
| S-Adenosylmethionine | 3.89E-05 | 1.61E-05 | 3.75E-05 | 1.72E-05 | 0.117 | 0.271 | 1.04 |
| D-Maltose | 9.87E-04 | 3.48E-04 | 9.09E-04 | 3.12E-04 | 0.118 | 0.271 | 1.09 |
| Muscomin | 3.23E-05 | 1.06E-05 | 3.02E-05 | 1.04E-05 | 0.122 | 0.278 | 1.07 |
| D-Serine | 3.12E-04 | 1.16E-04 | 2.89E-04 | 1.17E-04 | 0.127 | 0.286 | 1.08 |
| 1-Methylhypoxanthine | 6.03E-04 | 2.15E-04 | 5.67E-04 | 2.40E-04 | 0.129 | 0.289 | 1.06 |
| 5-HEPE | 1.58E-06 | 6.18E-06 | 1.45E-06 | 4.39E-06 | 0.139 | 0.307 | 1.09 |
| Quinone | 4.53E-03 | 3.77E-04 | 4.60E-03 | 3.44E-04 | 0.151 | 0.332 | 0.98 |
| Homoarecoline | 3.10E-05 | 8.85E-05 | 1.92E-05 | 1.03E-05 | 0.153 | 0.333 | 1.61 |
| Thiamine | 7.84E-05 | 5.84E-05 | 7.58E-05 | 6.28E-05 | 0.163 | 0.351 | 1.03 |
| Saccharopine | 6.66E-06 | 5.05E-06 | 7.35E-06 | 1.50E-05 | 0.165 | 0.352 | 0.91 |
| Creatinine | 3.33E-02 | 3.81E-03 | 3.41E-02 | 4.06E-03 | 0.176 | 0.362 | 0.98 |
| Pantothenic acid | 8.57E-04 | 3.81E-04 | 9.39E-04 | 3.90E-04 | 0.176 | 0.362 | 0.91 |
| Beta-D-Glucopyranuronic acid | 1.35E-05 | 5.97E-06 | 1.23E-05 | 5.84E-06 | 0.178 | 0.362 | 1.10 |
| 2-Propylpiperidine | 2.78E-04 | 3.27E-05 | 2.72E-04 | 3.30E-05 | 0.178 | 0.362 | 1.02 |
| Trimethylaminoacetone | 3.42E-05 | 1.33E-05 | 3.67E-05 | 1.46E-05 | 0.179 | 0.362 | 0.93 |
| Aminofructose 6-phosphate | 2.76E-05 | 1.33E-05 | 3.00E-05 | 1.45E-05 | 0.180 | 0.362 | 0.92 |
| Mesalazine | 7.21E-05 | 3.90E-05 | 7.81E-05 | 3.81E-05 | 0.181 | 0.362 | 0.92 |
| Lactaldehyde | 4.62E-05 | 1.98E-05 | 4.19E-05 | 1.71E-05 | 0.181 | 0.362 | 1.10 |
| Kynurenic acid | 2.11E-04 | 1.73E-04 | 2.03E-04 | 1.98E-04 | 0.183 | 0.363 | 1.04 |
| Thiamine monophosphate | 1.02E-05 | 4.00E-06 | 1.19E-05 | 4.72E-06 | 0.187 | 0.369 | 0.86 |
| 4-Hydroxy-2-butenoic acid gamma-lactone | 4.92E-04 | 6.27E-05 | 4.83E-04 | 1.01E-04 | 0.190 | 0.370 | 1.02 |
| N-Acetylputrescine | 3.76E-05 | 2.42E-05 | 3.31E-05 | 1.98E-05 | 0.194 | 0.375 | 1.14 |
| 2-Methylfuran | 2.12E-04 | 1.09E-04 | 1.94E-04 | 1.10E-04 | 0.195 | 0.375 | 1.09 |
| 1-Salicylate glucuronide | 2.09E-05 | 7.61E-06 | 2.01E-05 | 7.75E-06 | 0.214 | 0.408 | 1.04 |
| Guanine | 3.13E-04 | 1.50E-04 | 3.09E-04 | 2.03E-04 | 0.224 | 0.420 | 1.01 |
| Dihydroneopterin phosphate | 3.36E-05 | 1.37E-05 | 3.16E-05 | 1.46E-05 | 0.224 | 0.420 | 1.06 |
| 7-Aminoclonazepam | 9.01E-06 | 8.66E-06 | 7.07E-06 | 6.12E-06 | 0.227 | 0.424 | 1.28 |
| LysoPE(0:0/22:4(7Z,10Z,13Z,16Z)) | 1.37E-05 | 8.34E-06 | 1.25E-05 | 8.20E-06 | 0.231 | 0.428 | 1.09 |
| Gonyautoxin VI | 1.81E-04 | 6.52E-05 | 1.68E-04 | 6.40E-05 | 0.240 | 0.440 | 1.07 |
| L-Arginine | 2.81E-04 | 1.50E-04 | 3.01E-04 | 1.48E-04 | 0.247 | 0.446 | 0.93 |
| Diethanolamine | 8.12E-05 | 2.80E-05 | 9.39E-05 | 6.75E-05 | 0.248 | 0.446 | 0.87 |
| N-Methyl-a-aminoisobutyric acid | 1.78E-04 | 6.18E-05 | 1.72E-04 | 7.74E-05 | 0.248 | 0.446 | 1.03 |
| 5,6-Dihydroxyindole | 7.46E-05 | 2.80E-05 | 7.01E-05 | 2.86E-05 | 0.251 | 0.447 | 1.06 |
| Triglochinin | 1.10E-04 | 3.60E-05 | 1.04E-04 | 3.17E-05 | 0.254 | 0.450 | 1.06 |
| 2-Azetidinecarboxylic acid | 8.81E-04 | 2.11E-04 | 8.46E-04 | 1.93E-04 | 0.264 | 0.462 | 1.04 |
| 3,3,5-triiodo-L-thyronine-beta-D-glucuronoside | 1.66E-03 | 5.08E-04 | 1.77E-03 | 7.49E-04 | 0.265 | 0.462 | 0.93 |
| 5-Methylcytidine | 7.47E-05 | 1.76E-05 | 7.28E-05 | 2.14E-05 | 0.274 | 0.475 | 1.03 |
| L-4-Hydroxyglutamate semialdehyde | 1.34E-04 | 3.35E-05 | 1.30E-04 | 3.71E-05 | 0.290 | 0.500 | 1.03 |
| Ethenyl acetate | 3.38E-04 | 2.35E-05 | 3.42E-04 | 2.52E-05 | 0.303 | 0.515 | 0.99 |
| Valyl-Lysine | 6.21E-05 | 1.76E-05 | 5.98E-05 | 1.91E-05 | 0.305 | 0.515 | 1.04 |
| N1-(2-Hydroxyethyl)flurazepam | 1.86E-03 | 9.83E-04 | 1.74E-03 | 7.21E-04 | 0.305 | 0.515 | 1.07 |
| Epidermin | 3.79E-06 | 2.17E-06 | 4.30E-06 | 2.54E-06 | 0.322 | 0.540 | 0.88 |
| 1-Methylnicotinamide | 2.10E-04 | 7.26E-05 | 2.21E-04 | 8.33E-05 | 0.333 | 0.548 | 0.95 |
| Pyridine | 1.07E-04 | 2.85E-05 | 1.10E-04 | 2.57E-05 | 0.334 | 0.548 | 0.97 |
| Lacto-N-difucopentaose II | 7.53E-06 | 4.02E-06 | 7.55E-06 | 3.83E-06 | 0.334 | 0.548 | 1.00 |
| O-Acetylserine | 1.05E-02 | 2.10E-03 | 1.02E-02 | 2.29E-03 | 0.336 | 0.548 | 1.02 |
| Sorbose 1-phosphate | 2.78E-05 | 6.13E-06 | 2.68E-05 | 5.80E-06 | 0.341 | 0.552 | 1.04 |
| Phosphoribosyl formamidocarboxamide | 3.21E-05 | 6.18E-06 | 3.29E-05 | 6.29E-06 | 0.348 | 0.561 | 0.98 |
| Prolylhydroxyproline | 5.73E-05 | 2.20E-05 | 5.47E-05 | 2.28E-05 | 0.351 | 0.562 | 1.05 |
| Neotrehalose | 2.93E-03 | 3.42E-04 | 2.98E-03 | 3.64E-04 | 0.357 | 0.566 | 0.98 |
| N-Ornithyl-L-taurine | 4.50E-05 | 2.34E-05 | 5.38E-05 | 4.08E-05 | 0.359 | 0.566 | 0.84 |
| Uralenneoside | 2.87E-05 | 1.35E-05 | 2.67E-05 | 1.15E-05 | 0.361 | 0.566 | 1.07 |
| Turanose | 1.61E-03 | 6.34E-04 | 1.54E-03 | 5.35E-04 | 0.365 | 0.569 | 1.05 |
| 1-Amino-propan-2-ol | 9.56E-06 | 4.24E-06 | 9.20E-06 | 4.74E-06 | 0.370 | 0.573 | 1.04 |
| 3-Hydroxy-4-butanolide | 2.63E-05 | 6.52E-06 | 2.69E-05 | 5.80E-06 | 0.372 | 0.573 | 0.98 |
| 4-Amino-2-methylenebutanoic acid | 1.21E-04 | 5.60E-05 | 1.41E-04 | 9.41E-05 | 0.374 | 0.573 | 0.86 |
| (Â±)-erythro-Isoleucine | 6.88E-04 | 2.39E-04 | 7.29E-04 | 3.53E-04 | 0.385 | 0.585 | 0.94 |
| 2-Hydroxypyridine | 2.09E-04 | 2.48E-05 | 2.12E-04 | 2.88E-05 | 0.390 | 0.589 | 0.98 |
| Phenylacetaldehyde | 1.25E-05 | 3.27E-06 | 5.92E-05 | 4.25E-04 | 0.392 | 0.589 | 0.21 |
| 2,5-Dihydro-2,4-dimethyloxazole | 4.02E-03 | 2.94E-03 | 4.08E-03 | 3.82E-03 | 0.394 | 0.589 | 0.99 |
| 2'-Deoxyguanosine 5'-monophosphate | 1.00E-04 | 1.43E-05 | 9.89E-05 | 1.65E-05 | 0.406 | 0.603 | 1.01 |
| Creatine | 1.67E-02 | 2.34E-03 | 1.64E-02 | 1.95E-03 | 0.420 | 0.620 | 1.02 |
| LysoPE(18:1(9Z)/0:0) | 1.47E-04 | 8.84E-05 | 1.51E-04 | 1.17E-04 | 0.430 | 0.629 | 0.97 |
| 3-Oxohexadecanoyl-CoA | 6.35E-05 | 3.26E-05 | 6.44E-05 | 2.97E-05 | 0.431 | 0.629 | 0.99 |
| Allysine | 1.68E-04 | 1.32E-04 | 1.77E-04 | 2.43E-04 | 0.453 | 0.657 | 0.95 |
| 5-Methyl-2-furancarboxaldehyde | 5.87E-04 | 3.54E-04 | 5.61E-04 | 3.64E-04 | 0.467 | 0.673 | 1.05 |
| Triethanolamine | 7.59E-05 | 1.52E-04 | 1.09E-04 | 4.72E-04 | 0.470 | 0.673 | 0.70 |
| LysoPE(0:0/20:4(5Z,8Z,11Z,14Z)) | 4.00E-05 | 2.55E-05 | 4.09E-05 | 2.68E-05 | 0.490 | 0.697 | 0.98 |
| 4-Acetyl-3-methylpyridine | 7.85E-05 | 2.80E-05 | 8.33E-05 | 3.88E-05 | 0.492 | 0.697 | 0.94 |
| Chondroitin | 6.14E-05 | 1.74E-05 | 5.99E-05 | 1.63E-05 | 0.502 | 0.703 | 1.03 |
| 3'-Sialyllactose | 4.84E-05 | 1.00E-05 | 4.96E-05 | 1.15E-05 | 0.502 | 0.703 | 0.98 |
| 1-Methyladenosine | 3.00E-05 | 1.15E-05 | 3.20E-05 | 1.31E-05 | 0.517 | 0.720 | 0.94 |
| 3-Buten-2-one | 1.10E-04 | 9.63E-06 | 1.10E-04 | 1.26E-05 | 0.559 | 0.775 | 1.01 |
| D-4'-Phosphopantothenate | 4.54E-06 | 1.84E-06 | 4.93E-06 | 2.49E-06 | 0.582 | 0.801 | 0.92 |
| Methyl 2-furoate | 3.24E-04 | 1.20E-04 | 3.42E-04 | 1.60E-04 | 0.584 | 0.801 | 0.95 |
| 2-Aminoacrylic acid | 1.72E-05 | 1.04E-05 | 1.61E-05 | 1.06E-05 | 0.592 | 0.807 | 1.07 |
| alpha-Zearalenol | 4.93E-05 | 1.97E-05 | 4.87E-05 | 2.09E-05 | 0.595 | 0.807 | 1.01 |
| Adenosine 2'-phosphate | 2.43E-05 | 2.59E-05 | 2.45E-05 | 2.74E-05 | 0.603 | 0.811 | 0.99 |
| Glyceraldehyde | 2.71E-05 | 1.46E-05 | 2.97E-05 | 2.00E-05 | 0.606 | 0.811 | 0.91 |
| Lacto-N-fucopentaose III | 1.92E-05 | 1.14E-05 | 1.84E-05 | 8.53E-06 | 0.608 | 0.811 | 1.04 |
| 3alpha,4,5,7alpha-Tetrahydro-5-hydroxy-1H-isoindole-1,3(2H)-dione | 7.75E-04 | 3.81E-04 | 8.20E-04 | 4.25E-04 | 0.619 | 0.818 | 0.94 |
| Gerberinol | 8.70E-05 | 3.15E-05 | 8.38E-05 | 2.87E-05 | 0.620 | 0.818 | 1.04 |
| Dehydrophytosphingosine | 6.13E-06 | 6.68E-06 | 6.42E-06 | 7.02E-06 | 0.636 | 0.834 | 0.96 |
| Malondialdehyde | 1.43E-04 | 1.13E-05 | 1.42E-04 | 1.24E-05 | 0.656 | 0.856 | 1.00 |
| L-Phenylalanine | 1.09E-03 | 3.49E-04 | 1.09E-03 | 4.75E-04 | 0.669 | 0.857 | 1.00 |
| 3-Methylhistidine | 4.75E-04 | 5.52E-04 | 5.60E-04 | 7.66E-04 | 0.670 | 0.857 | 0.85 |
| Beta-Aminopropionitrile | 4.30E-05 | 1.00E-05 | 4.30E-05 | 7.48E-06 | 0.677 | 0.857 | 1.00 |
| Asparaginyl-Hydroxyproline | 5.72E-04 | 2.50E-04 | 6.51E-04 | 2.85E-04 | 0.677 | 0.857 | 0.88 |
| 4-Hydroxybenzaldehyde | 2.57E-05 | 1.35E-05 | 2.81E-05 | 1.83E-05 | 0.684 | 0.857 | 0.91 |
| Sphinganine | 5.16E-05 | 1.04E-04 | 3.67E-05 | 8.81E-05 | 0.685 | 0.857 | 1.41 |
| Benzyl cinnamate | 2.79E-05 | 4.63E-06 | 2.78E-05 | 5.21E-06 | 0.687 | 0.857 | 1.00 |
| 2-Acetyl-3,6-dimethylpyrazine | 3.18E-04 | 1.52E-04 | 3.38E-04 | 1.71E-04 | 0.688 | 0.857 | 0.94 |
| L-Valine | 5.74E-05 | 1.90E-05 | 5.68E-05 | 1.84E-05 | 0.692 | 0.857 | 1.01 |
| 6-Hydroxy-1H-indole-3-acetamide | 9.47E-05 | 1.95E-05 | 9.54E-05 | 1.80E-05 | 0.695 | 0.857 | 0.99 |
| trans-3,3',4',5,5',7-Hexahydroxyflavanone | 8.36E-05 | 4.01E-05 | 8.13E-05 | 3.46E-05 | 0.695 | 0.857 | 1.03 |
| Taurine | 3.32E-03 | 1.32E-03 | 3.37E-03 | 1.23E-03 | 0.699 | 0.858 | 0.99 |
| Prolyl-Aspartate | 2.56E-05 | 8.60E-06 | 2.59E-05 | 1.12E-05 | 0.705 | 0.861 | 0.99 |
| 1-Methyl-2-pyrrolecarboxaldehyde | 2.95E-05 | 8.15E-06 | 2.93E-05 | 8.24E-06 | 0.716 | 0.870 | 1.01 |
| L-Tyrosine | 2.28E-04 | 8.59E-05 | 2.39E-04 | 1.15E-04 | 0.725 | 0.877 | 0.95 |
| O-Phosphotyrosine | 1.08E-04 | 5.47E-05 | 1.04E-04 | 4.15E-05 | 0.733 | 0.880 | 1.04 |
| 3,4,5-Trimethoxycinnamic acid | 2.06E-05 | 1.13E-05 | 2.16E-05 | 1.96E-05 | 0.735 | 0.880 | 0.96 |
| Octadecanamide | 2.85E-06 | 1.48E-06 | 2.97E-06 | 2.48E-06 | 0.740 | 0.882 | 0.96 |
| L-Carnitine | 3.02E-02 | 2.37E-03 | 3.05E-02 | 2.96E-03 | 0.746 | 0.885 | 0.99 |
| 6-Chloro-N-(1-methylethyl)-1,3,5-triazine-2,4-diamine | 2.41E-04 | 2.36E-04 | 2.32E-04 | 2.81E-04 | 0.751 | 0.887 | 1.04 |
| Ornithine | 4.45E-05 | 3.63E-05 | 5.07E-05 | 5.35E-05 | 0.759 | 0.892 | 0.88 |
| L-Asparagine | 8.24E-05 | 4.15E-05 | 9.33E-05 | 7.45E-05 | 0.764 | 0.894 | 0.88 |
| Cellobiose | 1.55E-03 | 5.90E-04 | 1.52E-03 | 5.56E-04 | 0.770 | 0.897 | 1.02 |
| Yuccaol C | 5.39E-05 | 2.10E-05 | 5.28E-05 | 2.00E-05 | 0.779 | 0.902 | 1.02 |
| (2R)-2-Hydroxy-2-methylbutanenitrile | 1.79E-05 | 7.19E-06 | 1.86E-05 | 1.17E-05 | 0.782 | 0.902 | 0.96 |
| L-Glutamic acid | 1.33E-03 | 2.45E-04 | 1.36E-03 | 3.02E-04 | 0.789 | 0.906 | 0.98 |
| Proline betaine | 1.65E-05 | 1.29E-05 | 1.90E-05 | 2.65E-05 | 0.801 | 0.906 | 0.87 |
| Nicotine | 1.55E-05 | 1.15E-05 | 1.75E-05 | 3.36E-05 | 0.804 | 0.906 | 0.88 |
| TG(18:1(9Z)/16:0/22:5(7Z,10Z,13Z,16Z,19Z)) | 3.89E-06 | 1.13E-06 | 3.94E-06 | 1.23E-06 | 0.805 | 0.906 | 0.99 |
| Methoxyacetic acid | 3.25E-03 | 2.27E-04 | 3.25E-03 | 2.34E-04 | 0.809 | 0.906 | 1.00 |
| Tromethamine | 1.29E-04 | 2.15E-05 | 1.30E-04 | 2.54E-05 | 0.809 | 0.906 | 0.99 |
| 1-Kestose | 3.48E-04 | 1.25E-04 | 3.45E-04 | 1.26E-04 | 0.811 | 0.906 | 1.01 |
| 2-Furanmethanol | 1.55E-04 | 8.16E-05 | 1.53E-04 | 7.85E-05 | 0.818 | 0.910 | 1.02 |
| Propionylcarnitine | 1.36E-03 | 5.17E-04 | 1.43E-03 | 1.16E-03 | 0.849 | 0.938 | 0.96 |
| Cohibin A | 2.05E-06 | 1.42E-06 | 2.48E-06 | 3.70E-06 | 0.855 | 0.938 | 0.83 |
| L-Threonine | 4.94E-04 | 2.30E-04 | 4.76E-04 | 1.92E-04 | 0.855 | 0.938 | 1.04 |
| Piperidine | 4.56E-04 | 1.79E-04 | 4.59E-04 | 1.86E-04 | 0.868 | 0.948 | 0.99 |
| 2-Methyl-3-oxopropanoic acid | 2.82E-04 | 2.58E-05 | 2.83E-04 | 2.95E-05 | 0.872 | 0.948 | 1.00 |
| Bilirubin | 1.33E-05 | 1.23E-05 | 1.21E-05 | 9.71E-06 | 0.877 | 0.948 | 1.10 |
| 2,6-Dimethylpyrazine | 6.74E-05 | 3.27E-05 | 6.64E-05 | 3.47E-05 | 0.879 | 0.948 | 1.01 |
| 3-Amino-2-piperidone | 2.07E-04 | 9.60E-05 | 2.18E-04 | 1.26E-04 | 0.883 | 0.948 | 0.95 |
| Racemethionine | 2.84E-04 | 1.03E-04 | 2.94E-04 | 1.25E-04 | 0.892 | 0.953 | 0.97 |
| Millefin | 3.53E-05 | 1.24E-05 | 3.56E-05 | 1.28E-05 | 0.896 | 0.954 | 0.99 |
| 1,2,3,4-Tetrahydro-b-carboline-1,3-dicarboxylic acid | 4.67E-05 | 1.25E-05 | 4.67E-05 | 1.35E-05 | 0.916 | 0.971 | 1.00 |
| 3-Isoxazolidinone | 4.26E-05 | 1.69E-05 | 4.21E-05 | 1.54E-05 | 0.922 | 0.973 | 1.01 |
| (E)-2-Butenal | 1.62E-05 | 9.54E-06 | 1.62E-05 | 9.46E-06 | 0.942 | 0.989 | 1.00 |
| (Â±)-Tryptophan | 9.14E-05 | 2.77E-05 | 9.50E-05 | 5.24E-05 | 0.945 | 0.989 | 0.96 |
| FAPy-adenine | 2.01E-05 | 5.02E-06 | 2.02E-05 | 4.88E-06 | 0.955 | 0.991 | 0.99 |
| Choline | 3.53E-02 | 9.00E-03 | 3.56E-02 | 1.02E-02 | 0.958 | 0.991 | 0.99 |
| Spinacetin 3-gentiobioside | 2.28E-05 | 1.98E-05 | 2.24E-05 | 2.08E-05 | 0.963 | 0.991 | 1.02 |
| Acetone cyanohydrin | 1.51E-04 | 2.59E-05 | 1.51E-04 | 2.53E-05 | 0.967 | 0.991 | 1.00 |
| Phosphocreatine | 1.15E-04 | 1.92E-04 | 1.61E-04 | 2.74E-04 | 0.973 | 0.991 | 0.71 |
| Protoanemonin | 3.42E-04 | 1.47E-04 | 3.64E-04 | 2.36E-04 | 0.979 | 0.991 | 0.94 |
| D-Galactose | 9.53E-03 | 6.34E-04 | 9.55E-03 | 8.09E-04 | 0.982 | 0.991 | 1.00 |
| N6-Methyladenosine | 6.98E-04 | 9.46E-05 | 7.02E-04 | 1.14E-04 | 0.984 | 0.991 | 0.99 |
| N6-Carbamoyl-L-threonyladenosine | 2.45E-06 | 7.05E-07 | 2.51E-06 | 1.32E-06 | 0.985 | 0.991 | 0.97 |
| Leukoaminochrome | 4.32E-05 | 5.55E-06 | 4.34E-05 | 6.48E-06 | 0.987 | 0.991 | 1.00 |
| 2,5-Dimethyloxazole | 3.06E-04 | 1.42E-04 | 3.06E-04 | 1.41E-04 | 0.995 | 0.995 | 1.00 |

Table S9 The concentrations of macro and micro-elements for all analyzed samples over the entire measurement period in the three clusters

| ion | unit | mean_1 | mean_2 | mean_3 | sd_1 | sd_2 | sd_3 | p_anova | p_adj |
| --- | --- | --- | --- | --- | --- | --- | --- | --- | --- |
| Al | g/kg | 0.001 | 0.001 | 0.001 | 0.001 | 0.001 | 0.001 | 0.000 | 0.000 |
| As | mg/kg | 0.002 | 0.002 | 0.002 | 0.001 | 0.001 | 0.001 | 0.000 | 0.000 |
| B | mg/kg | 0.407 | 0.562 | 0.387 | 0.135 | 0.132 | 0.100 | 0.000 | 0.000 |
| Ba | mg/kg | 0.029 | 0.025 | 0.022 | 0.019 | 0.013 | 0.010 | 0.000 | 0.000 |
| Ca | mg/kg | 349.999 | 313.715 | 279.367 | 77.413 | 98.186 | 45.327 | 0.000 | 0.000 |
| Cd | μg/kg | 0.322 | 0.414 | 0.202 | 0.194 | 0.176 | 0.158 | 0.000 | 0.000 |
| Co | mg/kg | 0.002 | 0.003 | 0.002 | 0.001 | 0.001 | 0.001 | 0.000 | 0.000 |
| Cr | mg/kg | 0.153 | 0.286 | 0.134 | 0.064 | 0.136 | 0.087 | 0.000 | 0.000 |
| Cu | mg/kg | 0.182 | 0.246 | 0.259 | 0.063 | 0.072 | 0.043 | 0.000 | 0.000 |
| Fe | mg/kg | 2.655 | 3.131 | 2.025 | 0.824 | 1.358 | 0.661 | 0.000 | 0.000 |
| Hg | μg/kg | 2.186 | 2.391 | 1.764 | 1.306 | 0.827 | 0.330 | 0.000 | 0.000 |
| K | mg/kg | 510.065 | 500.792 | 276.109 | 145.562 | 138.503 | 88.418 | 0.000 | 0.000 |
| Mg | mg/kg | 27.252 | 24.541 | 22.688 | 6.357 | 6.296 | 5.864 | 0.000 | 0.000 |
| Mn | mg/kg | 0.035 | 0.074 | 0.041 | 0.013 | 0.034 | 0.016 | 0.000 | 0.000 |
| Mo | mg/kg | 0.017 | 0.039 | 0.029 | 0.009 | 0.012 | 0.013 | 0.000 | 0.000 |
| Na | mg/kg | 97.020 | 120.843 | 79.618 | 30.772 | 63.086 | 25.403 | 0.000 | 0.000 |
| Ni | mg/kg | 0.043 | 0.076 | 0.046 | 0.022 | 0.040 | 0.024 | 0.000 | 0.000 |
| Pb | mg/kg | 0.032 | 0.039 | 0.032 | 0.030 | 0.014 | 0.015 | 0.000 | 0.000 |
| Sb | mg/kg | 0.000 | 0.001 | 0.001 | 0.000 | 0.001 | 0.001 | 0.000 | 0.000 |
| Se | mg/kg | 0.009 | 0.048 | 0.010 | 0.006 | 0.030 | 0.008 | 0.000 | 0.000 |
| Sn | mg/kg | 0.008 | 0.011 | 0.006 | 0.007 | 0.005 | 0.004 | 0.000 | 0.000 |
| Sr | mg/kg | 0.056 | 0.090 | 0.062 | 0.019 | 0.032 | 0.020 | 0.000 | 0.000 |
| Ti | mg/kg | 0.244 | 0.425 | 0.252 | 0.084 | 0.118 | 0.094 | 0.000 | 0.000 |
| Tl | μg/kg | 0.047 | 0.055 | 0.043 | 0.054 | 0.045 | 0.037 | 0.000 | 0.000 |
| V | mg/kg | 0.006 | 0.009 | 0.008 | 0.002 | 0.002 | 0.002 | 0.000 | 0.000 |
| Zn | mg/kg | 0.607 | 1.930 | 1.215 | 0.352 | 1.040 | 0.570 | 0.000 | 0.000 |


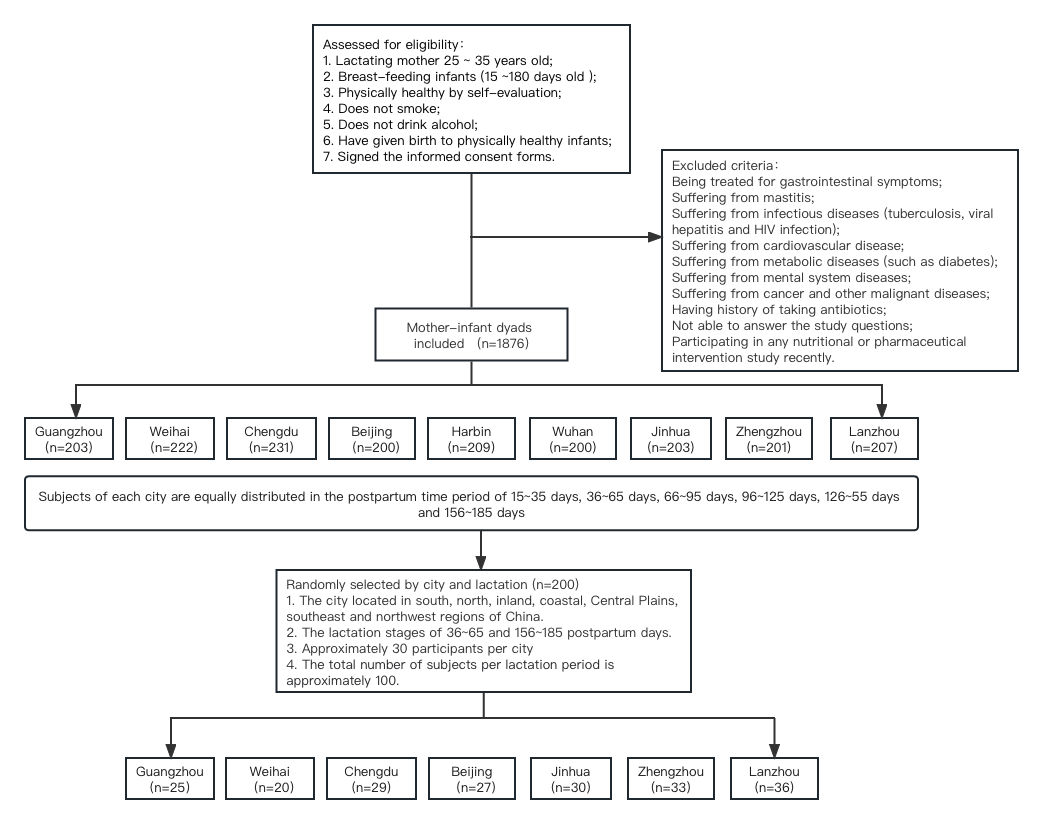


Supplementary Figure 1. The descriptions of inclusion/exclusion criteria and participants flow chart
